# An assessment of forest biomass maps in Europe using harmonized national statistics and inventory plots

**DOI:** 10.1016/j.foreco.2017.11.047

**Published:** 2018-02-01

**Authors:** Valerio Avitabile, Andrea Camia

**Affiliations:** European Commission, Joint Research Centre, Via E. Fermi 2749, 21027 Ispra, Italy

**Keywords:** NFI, Forest inventory, Remote sensing, Aboveground biomass, Forest plot, Carbon cycle

## Abstract

Maps of aboveground forest biomass based on different input data and modelling approaches have been recently produced for Europe, opening up the possibility for several applications and products not obtainable by summary statistics. However, the accuracy assessment of the existing maps is limited by the lack of reference data consistent over the study region and representative of the maps cells. Here, we used harmonized forest biomass data for 26 European countries derived by National Forest Inventories using a common biomass definition and estimator to assess four biomass maps. The assessment was performed at regional, national and sub-national scales using harmonized statistics derived from almost half million ground plot measurements, and at pixel level using a subset of 22,166 plots covering most European forest types. The field plots were temporally aligned with the maps using growth rates and further screened using an innovative approach based on tree cover variability to remove the plots not representative of the map cells. The harmonized reference data showed that all maps tended to overestimate at low biomass (<100 Mg ha^−1^) and underestimate at medium – high biomass (>100 Mg ha^−1^), resulting in an overall negative bias (23–43 Mg ha^−1^ at national level) relative to the harmonized estimates. The maps relative errors ranged from 29% to 40% at national level and increased at higher resolutions, reaching 58–67% at pixel level. We also assessed the effect of the harmonization of the national statistics and report that the harmonized biomass values present significant differences compared to the national estimates for 14 countries, and provide a slightly higher stock (+3.8%) at European scale. We show that harmonized and representative reference data are essential to properly assess the accuracy of biomass maps, and we further identify the factors affecting the maps performance and provide indications for their improvements.

## Highlights

• We assessed four biomass maps for Europe using harmonized biomass reference data.
• The harmonized statistics were derived from ∼430,000 plots from 26 countries.
• All maps overestimated at low biomass and underestimated at high biomass.
• All maps had an overall negative bias (23–43 Mg ha^−1^ at national level).
• The maps relative errors was 29–40% at national level and 63–72% at cell level.

## 1 Introduction

Forests cover 38% of the land area in the European Union (EU) and provide key environmental functions and socio-economic benefits (FOREST EUROPE, 2015). Updated and harmonized spatially-explicit estimates of the forest biomass stocks in Europe are necessary to support the EU policies on bioeconomy and renewable resources, as well as to improve climate change modelling and design appropriate mitigation actions (EC, 2011, Grassi et al., 2017, Nabuurs et al., 2013, Scarlat et al., 2015).

Most European countries have a National Forest Inventory (NFI) providing reliable statistics on aboveground dry biomass of living trees in forest areas (hereafter, biomass) at national scale (Tomppo et al., 2010, Vidal et al., 2016). However, the NFI data are not always recent or frequently updated, often do not provide the spatial distribution of biomass, and are based on country-specific inventory designs and biomass definitions that make their integration difficult for a regional (i.e., European) assessment of biomass resources (Lawrence et al., 2010, McRoberts et al., 2010, Neumann et al., 2016).

During the last decade and mostly independently from the NFIs, a few biomass maps have been produced at European or global scales using different input data and modelling approaches (Barredo et al., 2012, Gallaun et al., 2010, Kindermann et al., 2008, Thurner et al., 2014). These maps provide wall-to-wall biomass estimates over forested areas, but the level of reliability of their estimates is difficult to assess since the remote sensing signals used for the estimations are only indirectly related to the biomass density of vegetation. Moreover, the maps validation is limited by the lack of reference data consistent over the study region and with a spatial resolution comparable to that of the map cells (Hill et al., 2013, Mitchard et al., 2013, Réjou-Méchain et al., 2014).

Hence, there is a need to assess the coherence between “top-down” data from global or regional biomass maps produced by the remote sensing community with “bottom-up” statistics and plots from the NFIs, which are recognized as official estimates at the national and international levels such as for the reporting to the FAO and to the UN Framework Convention on Climate Change (Avitabile et al., 2011, Hill et al., 2013, Moreno et al., 2016).

In this study, we use harmonized biomass statistics and field plots for 26 European countries derived by NFI organizations in the context of service contracts launched by the Joint Research Centre (JRC) of the European Commission using a common biomass definition and estimator. This dataset is used to assess the uncertainties of four recently published biomass maps produced with varying degrees of complexity, from simple spatialization of average values using forest and ecozone maps to calibration of satellite and ancillary data using statistical models. Given the large differences in spatial and temporal resolutions between the reference plots (<1 ha) and the map cells (∼1 km^2^), an automated screening procedure is implemented to remove the plots not representative of the pixels. This study aims to better understand the capabilities and limitations of existing datasets and related methods, providing important information towards an improved mapping of forest biomass in Europe.

## 2 Material and methods

### 2.1 Harmonized biomass data

National statistics on forest biomass based on NFI data are periodically reported for all European countries by the national authorities for regional and global assessment purposes, such as the FAO Forest Resource Assessment (FRA) initiative (FAO, 2015) and the State of Europe’s Forest (FOREST EUROPE, 2015). Furthermore, some European countries provide online access to biomass statistics at sub-national levels. However, the biomass data provided by different countries are not directly comparable because they employ: (i) different definitions (i.e., refer to different biomass pools); (ii) different approaches to estimate biomass from the tree parameters (i.e., allometric equations or biomass conversion and expansion factors); (iii) different sampling designs and estimators to compute biomass stock over the study area; (iv) different timeframes as each NFI refers to a specific time period (Tomppo et al., 2010).

**Fig. 1:**
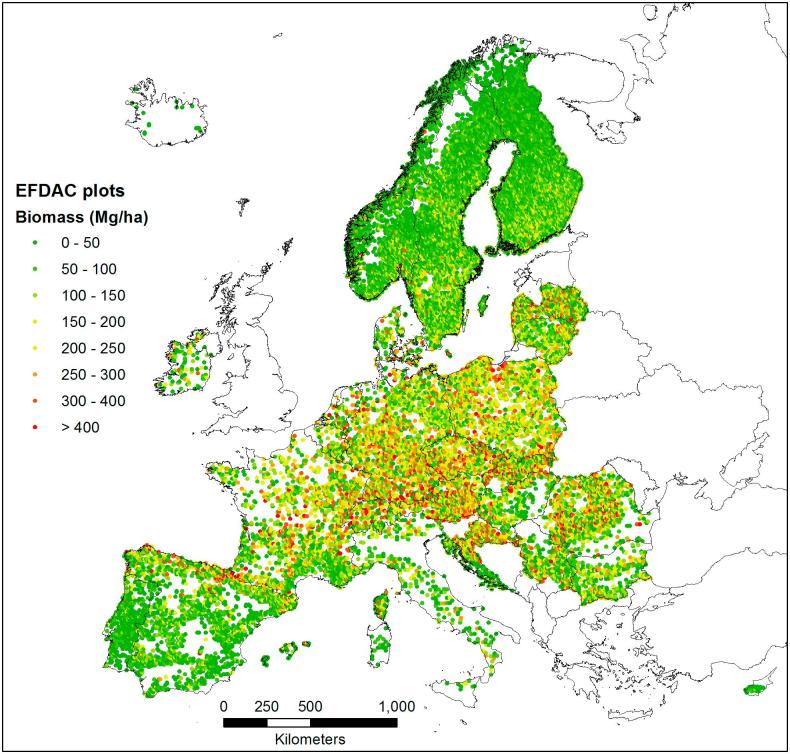
Spatial distribution of the harmonized EFDAC biomass plots for the 26 countries included in the EFDAC dataset.

Given the need of harmonized forest biomass statistics in Europe, the JRC launched two service contracts in 2013 and 2015 on the “Use of National Forest Inventory data to estimate biomass in the European Forests”. These contracts aimed to identify and apply a common biomass definition and estimator and obtain harmonized and comparable biomass estimates at national and sub-national levels for 26 European countries (Henning et al., 2016, Korhonen et al., 2014). The service contracts were implemented in the context of the JRC “Framework Contract for the provision of forest data and services in support of the European Forest Data Centre (EFDAC)”, which was also used to derive a harmonized dataset of tree species occurrence for Europe now publicly available (Mauri et al., 2017). Hereafter, the harmonized biomass estimates derived by the 26 participating NFIs organizations (see Acknowledgments) will be shortly referred to as the harmonized EFDAC biomass dataset. The 26 countries included in this study are shown in Fig. 1.

The harmonized definition included all aboveground biomass compartments of living trees, namely the aboveground part of the stump, the stem from stump to top, dead and living branches, and foliage. The common estimator was a design-based unbiased estimator applicable anywhere in Europe regardless of the stratification, point weighting and use of clusters in the original NFI sampling scheme (Lanz, 2012). Biomass was estimated for the areas defined as forest according to the FAO reference definition (FAO, 2000).

#### 2.1.1 Harmonized biomass statistics

The harmonized EFDAC biomass statistics consist of the total biomass stock and its sampling error (in units of Mg), the mean biomass density and its sampling error (Mg ha^−1^), and the forest area where biomass is estimated (ha). These values were calculated at national level and at the sub-national levels corresponding, for most countries, to the 2010 Classification of Territorial Units for Statistics (NUTS) level 2.

The 26 countries provided four different estimates of the total and mean biomass, obtained using the national or the harmonized definition of biomass in combination with the national or the common estimator. These estimates were obtained from a total of 431,261 field plots located in a forest area of 156 million ha, and were provided for individual species and for species groups (broadleaves and coniferous).

In the present study, the EFDAC biomass statistics were compiled, screened for errors, checked for consistency with published statistics (FAO, 2015, FOREST EUROPE, 2015), and analyzed. Seven countries did not report the sampling error related to the national estimator and in this study it was assumed equal to that of the common estimator. The statistics based on the harmonized definition and common estimator were then used as reference values for the assessment of the biomass maps.

#### 2.1.2 Harmonized biomass plots

Almost half million ground measurements within forest land have been acquired in Europe by several NFIs during the last two decades. However, most of the plot measurements are not accessible to researchers outside the national authorities for privacy reasons. Within the mentioned JRC service contracts, the 26 participating organizations made available a systematic subset of the NFI plots, providing the biomass density according to the harmonized definition and common estimator of one NFI plot for each 8 km INSPIRE grid cell. The plot nearest to the centre of the 8 km cell was selected and provided with a geolocation approximated to the 1 km INSPIRE grid. In total, the subset included 22,166 field plots with an almost complete spatial coverage of the European forests (Fig. 1).

### 2.2 Biomass maps

#### 2.2.1 Maps description

**Fig. 2:**
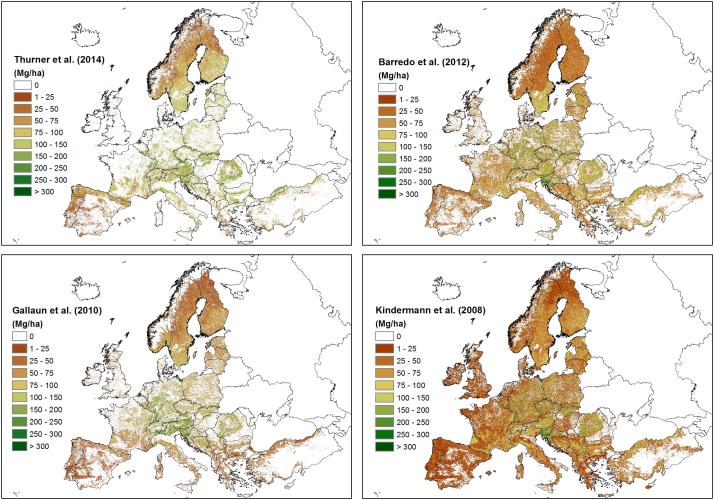
The four biomass maps for Europe: Thurner, Barredo, Kindermann and Gallaun (clockwise, from upper left).

Currently, there are four published maps providing forest biomass density for Europe: the datasets of Barredo et al., 2012, Kindermann et al., 2008, Gallaun et al., 2010, Thurner et al., 2014 (Fig. 2). These maps are hereafter referred to with the name of the first author. An overview of the map characteristics is provided in Table 1.

**Table 1:** Main characteristics of the four biomass maps for Europe.

Map	Barredo	Kindermann	Gallaun	Thurner
Calibration data	IPCC Tier 1	FRA 2010	NFI plots	NFI stats
Spatial data	CORINE, GEZ	MODIS NPP	MODIS data, VCF	ASAR
Auxiliary data	FRA 2010	Human impact map	CORINE, EFISCEN	BECF, GLC2000
Forest mask	CORINE	GLC2000 (>20%)	CORINE, FRA 2000	GLC2000 (>50%)
Year	2010	2010	2000	2010
Resolution	1 km	0.0083°	500 m	0.01°

The Barredo map spatializes the IPCC Tier 1 biomass density values per forest type (broadleaves, coniferous) and ecozone using the CORINE Land Cover 2006 map (Bossard et al., 2000) and the FAO Global Ecological Zone (GEZ) map (FAO, 2001), following an approach similar to that presented by Reusch and Gibbs (2008). In addition, the Barredo map further post-adjust the estimates at pixel level by applying ratios to match the national values reported in the FAO FRA 2010 (FAO, 2010). This map provides biomass and carbons stock density for Europe at 1 km resolution for the year 2010.

The Kindermann map downscales the FAO FRA 2010 national biomass statistics using the MODIS Net Primary Production (NPP) annual products (Cramer et al., 1999) and a map of human impact (CIESIN, 2002), assuming biomass linearly related to NPP and inversely related to human influence (Keeling and Phillips, 2007). This map provides biomass density globally at 0.0083° resolution. The map used in this study is the 2010 update (Kindermann, pers. comm.) of the map published in Kindermann et al. (2008), which was based on the FRA 2005 statistics.

The Gallaun map is based on the CORINE Land Cover 2000 map (Bossard et al., 2000), meteorological data (Hijmans et al., 2005) and MODIS images and Vegetation Continuous Field (VCF) products (Hansen et al., 2003), calibrated using NFI plot data from 16 European countries (Nabuurs et al., 2010). The volume estimates were further post-adjusted using correction ratios to match the regional EFISCEN data on growing stock in 2000 (Schelhaas et al., 2007) and converted to biomass using mean regional Biomass Expansion and Conversion Factors (BECFs) for broadleaves and conifers. This map provides growing stock volume at 500 m and biomass density at 10 km resolution for Europe for the year 2000. The map estimates are limited to the maximum value of 300 m^3^ ha^−1^ (approx. 256 Mg ha^−1^).

The Thurner map is based on the 2010 growing stock volume map derived from ENVISAT ASAR images (Santoro et al., 2011), converted to total forest carbon stock using BECFs and carbon fractions per leaf type derived from existing wood density databases and allometric relationships between biomass compartments. The Global Land Cover 2000 (GLC2000) map was used to identify the leaf types. This map provides aboveground forest carbon density of the northern hemisphere above 30° N for the year 2010 at 0.01° resolution.

The maps present an increasing level of complexity in their modelling approaches. A key difference among the maps is that the Barredo and Kindermann maps essentially downscale the total national biomass stocks using spatial data (hence, maintaining the correspondence with the total country values) while the Gallaun and Thurner maps used reference biomass data (plots or statistics) to calibrate an empirical model based on satellite images and auxiliary data. The Thurner map did not constrain the estimates to match the national statistics while the Gallaun map was ex-post adjusted to match the regional EFISCEN data for the regions covered by the model.

The maps also differed with regard to their spatial coverage because they used different forest masks. The Kindermann and Thurner maps used the forest classes of the GLC2000 map, which applies a minimum canopy cover of 15%. Both maps included the mosaic class where forest covers at least 50% of the pixel area, and the Kindermann map further included the mosaic class with a forest coverage of 20%. Instead, the Barredo and Gallaun maps used the forest areas of the CORINE land cover map, which applies a minimum canopy cover of 30%, but the Gallaun map further adjusted the forest cover threshold separately for each country to match the FRA 2000 statistics (Table 1).

#### 2.2.2 Maps pre-processing

The four biomass maps were first harmonized to the same spatial resolution (1 km), reference system (Lambert Azimuthal Equal Area), study area (Europe) and unit (aboveground biomass density in Mg ha^−1^) (Fig. 2).

The Gallaun map provided growing stock volume at 500 m and biomass density at 10 km resolution and it was harmonized using the following procedure. The volume map was first aggregated to 10 km and compared to the biomass map to derive BECFs for each 10 km cell, which were applied to convert the 500 m volume map to biomass units, and then aggregated to the resolution of 1 km.

The Thurner map was provided in Mg of carbon for the aboveground biomass compartments (stem, branches, and foliage), which were obtained using carbon fractions dependent on the leaf-type (broadleaf, needleleaf, mixed) as mapped by the GLC2000 map, and it was converted back to biomass units using the respective forest types according to the GLC2000 map.

### 2.3 Comparing the maps with the statistics

The four biomass maps were assessed by comparing them with the harmonized EFDAC biomass statistics for 26 European countries at regional, national and sub-national levels with regard to total biomass stocks and mean biomass densities. The sub-national level usually corresponded to the NUTS-2 administrative units, with the exception of few cases where only data at NUTS-1 level were provided.

The maps were also assessed with regard to the total forest area of the 26 countries, which was computed at the original map resolution after re-projection to the Lambert equal area reference system to avoid errors due to spatial aggregation effects.

The EFDAC statistics provide biomass density with regard to the forest area, while the biomass maps provide the biomass density relative to the land area because, due to their coarse resolution (∼1 km), the map cells often cover heterogeneous areas with only a fraction of forest cover. Thus, the mean biomass density of the maps for each administrative unit was computed after applying a common forest mask (i.e., setting to no data the map cells located in non-forest areas), in order to compare the mean densities of the maps and of the EFDAC statistics relative to the same forest areas. Instead, the biomass stock of the maps was comparable with the EFDAC data and it was computed by simply summing the stocks over the complete NUTS area without applying the forest mask.

The EFI Forest Map of Europe (Gunia et al., 2012), which provides the percent forest cover at 1 km resolution, was selected as forest mask because it was calibrated with the NFI statistics on forest area. The EFI map was converted to a forest – non forest map by selecting the cells with predominance of forest cover (i.e., larger than 50%). Such threshold produced a forest mask that was highly consistent with the forest area reported by the harmonized EFDAC datasets (i.e., linear correlation of 0.99 at national level).

### 2.4 Comparing the maps with the field plots

Besides the comparison at aggregated scales, the biomass maps were also assessed at pixel level as the local estimates and their spatial distribution are the real benefit of a biomass map compared to the NFI statistics. The plots were provided with geolocation approximated to the centre of the 1 km INSPIRE grid cell, and the biomass maps were resampled to the INSPIRE grid using bilinear interpolation before their comparison with the plots.

The maps estimates at pixel level were assessed relative to the harmonized EFDAC plot database by computing the bias (defined as the mean difference between the map and the plot values), the coefficient of determination of linear regression (r^2^), the Root Mean Square Error (RMSE), and the relative RMSE (defined as the RMSE divided by the mean plots value). These statistics were computed for all data pooled and per biomass class using bins of 100 Mg ha^−1^ defined with regard to the plot values. Assessing the error structure by biomass bins is essential for the users interested in the biomass density in contexts or forest types having specific biomass values, as a map can be unbiased over the complete biomass range but it can be heavily biased, and with different magnitudes, at certain biomass ranges.

Before comparison, given the spatial and temporal differences, the plots were screened and harmonized to the maps using the following procedure. In addition, the comparison was also performed using all available plots to assess the impact of the selection procedure.

#### 2.4.1 Temporal harmonization

The NFI field plots were measured in a range of several years (from 2002 to 2013) that in most cases did not match the reference year of the biomass maps (Table 1). For this reason, the biomass density of the plots was updated to the reference year of the maps using the IPCC Tier 1 growth rates (IPCC, 2006), increasing or decreasing the plot biomass of the increment occurred between the year of measurement of the plot and the reference year of the map. Since the IPCC increments are provided by continent and ecozone, each plot was related to an ecozone using the FAO GEZ 2010 map (FAO, 2012). In case the IPCC provided a range of values for the same ecozone, the average increment was applied. When the IPCC increment was related to the forest age (i.e., younger or older than 20 years), the appropriate value was chosen comparing the plot biomass with the expected biomass density at 20 years, which was in turn estimated using the increment reported for younger forest.

#### 2.4.2 Plot selection

The spatial mismatch between the resolution of the biomass maps and the field plots is very large, as the plots cover an area smaller than 1% of the maps cells. Hence, the field data needed to be screened to remove the plots not representative of the biomass density of the 1 km pixels (i.e., the plots located in contexts different from the average forest conditions of the pixels).

The representativeness of the plots was assessed based on the tree cover variability within the 1 km cells. The tree cover statistics were obtained from the 30 m Global Forest Cover dataset for the year 2000 (Hansen et al., 2013) and the related version for the year circa 2010 (USGS, 2016).

The plot representativeness was assessed according to two criteria. The first criterion excluded the plots in 1 km cells with heterogeneous tree cover, assuming that the biomass density would also vary accordingly. The 1 km cells were considered heterogeneous when the standard deviation of tree cover was larger than 15%. The second criterion further removed the plots not representative because of local scale variability not captured in the first criterion, i.e., the plots located in small patches of forest within a mainly non-forested pixel (i.e., plots with very high biomass in pixels with very low tree cover) or those in a small opening within a forested pixel (i.e., plots with very low biomass in pixels with very high tree cover). Those situations were identified using the relation between tree cover and biomass, which presents a positive correlation until the tree cover saturates. This relation was modelled using a logarithmic regression model with intercept at zero, and the plots with biomass outside of the 75% prediction interval of the regression function were removed.

The thresholds for the two criteria above were defined by combining visual analysis of Google Earth images, empirical testing and sensitivity analysis. For example, it was noted that restricting the threshold of the first criteria (i.e., the maximum tree cover standard deviation in a cell) from 15% to 10% reduced considerably the amounts of selected plots but did not change the assessment results, which however become less stable due to the small number of selected plots. Instead, increasing the tree cover standard deviation to values higher than 15% resulted in selecting pixels where the tree cover variability, assessed with visual analysis, became too large to assume plot representativeness.

In addition, plots with extremely high biomass were removed from the selected dataset, as it is unlikely that such values occur consistently over larger areas (i.e., 1 km^2^). According to the boxplot distribution of the complete dataset, the plots with biomass >400 Mg ha^−1^ were identified the outliers, as they exceeded the upper quartile by 1.5 times of the inter-quartile range, corresponding to about 2% of the complete dataset.

Lastly, the histogram of the selected plots was compared with that of the complete dataset to assess if the plot subset maintained a similar distribution of biomass values. The histogram of the selected plots tended to be skewed right, likely because the selection criteria tended to favour plots located in areas with closed canopy and therefore high biomass, while removing plots in open forests that are inherently characterized by large tree cover variability. To avoid bias in the assessment results, the selected plots were further pruned until their frequency distribution was similar to that of the unfiltered dataset. To do so, the plots were assessed by biomass bins of 50 Mg ha^−1^, and when the relative frequency of the screened plots was higher than that of the original dataset, a random sample was taken to match the relative frequency of the original dataset. This process was repeated iteratively until the relative difference among the selected and original plots for each biomass bin was smaller than 5%.

## 3 Results

### 3.1 Comparison of harmonized and national statistics

The analysis of the harmonized EFDAC statistics provided by Korhonen et al., 2014, Henning et al., 2016 showed that the total forest biomass stock estimated for the 26 European countries using the harmonized definition and common estimator was 3.8% higher than the value based on the national definitions and estimators (Table 2). The difference due only to the type of estimator was negligible (<0.4%) while the biomass definition was more relevant, with a stock increase of 4.1% when applying the harmonized definition compared to the national ones (using the national estimators). This is due to the fact that several countries use a national definition that does not include all aboveground biomass compartments, such as leaves or stumps, which are considered in the harmonized definition.

**Table 2:** Total forest biomass stock of 26 European countries using the national or harmonized definitions in combination with the national or common estimators.

Total biomass stock (Tg)			
	National definition	Harmonized definition	Difference (%)
National estimator	16,234	16,907	4.1%
Common estimator	16,213	16,846	3.9%
Difference (%)	−0.13%	−0.36%	3.8%

At country level, the total biomass computed using the harmonized definition and common estimator was significantly (i.e., compared to the sampling error of the estimates) higher than the value based on the national definitions and estimators for 11 countries (AT, BG, CH, DK, ES, FR, HR, HU, PT, RO, SE), smaller for 3 countries (BE, IE, IT), while no significant difference was found for the remaining 12 countries (CY, CZ, DE, FI, IS, LV, LT, NL, NO, PL, RS, SK). Conversely, only 4 countries (HR, DK, PT, SE) presented significant differences when comparing the stock estimated with the national and common estimators (using the harmonized definition in both cases), which confirmed the reliability of the common estimator to properly integrate samples obtained with different (country-specific) sampling designs. The results of total and mean biomass for each country are reported in the Supplementary Information (Fig. A.1 and A.2).

### 3.2 Maps assessment using the harmonized statistics

#### 3.2.1 Total biomass stock

The four biomass maps presented substantial differences in their estimates of biomass stock for the 26 European countries and showed a negative bias compared to the harmonized EFDAC statistics.

At regional level (26 countries), the stock estimated by the Barredo and Kindermann maps (16.6 and 16.4 Pg, respectively) matched closely the harmonized EFDAC statistics (16.8 Pg) while the Gallaun and Thurner maps provided lower estimates (13.0 and 14.2 Pg, respectively) (Table 3). Interestingly, the biomass estimates were not correlated to the forest area considered by the maps, since both the Barredo and Gallaun maps used the CORINE land cover map and covered a forest area (about 143 Mha) lower than the harmonized statistics (156 Mha), while the Kindermann and Thurner maps used the GLC2000 map and covered a higher forest area (about 174 Mha) (Table 3).

**Table 3:** Total forest area and biomass stock estimated by the biomass maps for the 26 European countries, and reference values from the harmonized EFDAC statistics.

Dataset	Forest area (1000 ha)	Biomass stock (Tg)
Barredo et al. (2012)	143,414	16,637
Kindermann et al. (2008)	174,401	16,400
Gallaun et al. (2010)	143,550	12,956
Thurner et al. (2014)	173,043	14,216
EFDAC statistics	156,134	16,846

**Fig. 3:**
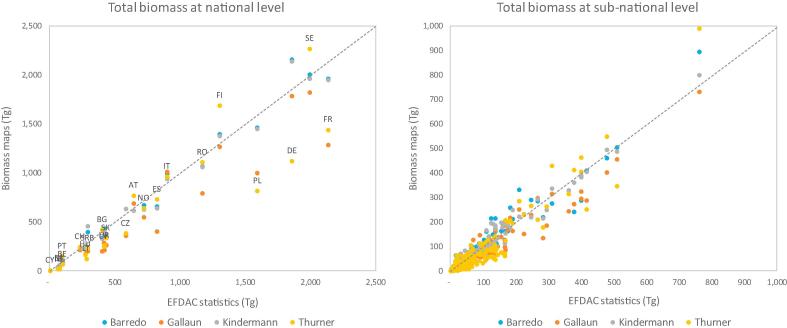
Comparison of the biomass stocks estimated by the biomass maps with the EFDAC statistics at national (left) and sub-national (right) levels.

At national and sub-national levels (305 units), the Barredo and Kindermann maps still matched well the EFDAC statistics, achieving smaller relative errors (14–15% at national level and 37–28% at subnational level, respectively) and small negative biases, while the Gallaun and Thurner maps presented larger errors (39% and 43% at national level and 48–56% at subnational level, respectively) and considerable negative bias (Fig. 3, Table 4).

**Table 4:** Assessment of the biomass stocks estimated by the biomass maps, obtained by comparing the maps with the EFDAC statistics at national and sub-national levels.

	Barredo	Gallaun	Kindermann	Thurner
	*National (NUTS 0)*			
Bias (Tg)	−8.1	−149.6	−17.2	−101.2
r^2^	0.98	0.91	0.98	0.83
RMSE (Tg)	93.6	253.6	97.2	279.9
Rel RMSE (%)	14%	39%	15%	43%

	*Sub-national (NUTS 2)*			
Bias (Tg)	−0.7	−13.3	−1.6	−9.6
r^2^	0.95	0.93	0.97	0.89
RMSE (Tg)	21.8	28.3	16.4	33.3
Rel RMSE (%)	37%	48%	28%	56%

The close correspondence of the Barredo and Kindermann maps with the reference values was because these maps directly used the FRA data to calibrate their estimates, which are closely related to the EFDAC statistics as they are both based on the same NFI data. It is noteworthy that the methods used by the Barredo and Kindermann maps to spatialize the total national values hold also at sub-national level, where they achieved better agreement with the reference values.

#### 3.2.2 Mean biomass density

**Fig. 4:**
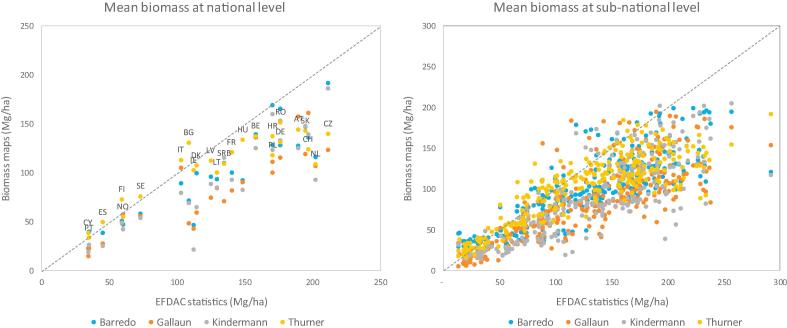
Comparison of the mean biomass densities estimated by the biomass maps and the EFDAC statistics at national (left) and sub-national (right) levels.

The comparison of the mean biomass density at national and sub-national levels, computed using the common forest mask, showed that all maps tended to underestimate biomass density compared to the harmonized EFDAC statistics (Fig. 4, Table 5). The relative accuracy was higher at national than at sub-national level, most likely because of aggregation effects, meaning that areas with overestimation and underestimation tended to compensate each other with an effect that increased with the size of the area considered (Moreno et al., 2016).

**Table 5:** Assessment of the mean biomass densities estimated by the biomass maps, obtained by comparing the maps with the EFDAC statistics at national and sub-national levels.

	Barredo	Gallaun	Kindermann	Thurner
	*National (NUTS 0)*			
Bias (Mg ha^−1^)	−29.9	−42.8	−37.7	−22.7
r^2^	0.83	0.77	0.79	0.79
RMSE (Mg ha^−1^)	37.5	50.5	45.0	36.2
Rel RMSE (%)	30%	40%	36%	29%

	*Sub-national (NUTS 2)*			
Bias (Mg ha^−1^)	−30.5	−43.9	−41.6	−29.1
r^2^	0.71	0.66	0.68	0.73
RMSE (Mg ha^−1^)	45.6	56.9	54.2	51.5
Rel RMSE (%)	36%	45%	43%	41%

The lowest relative error was achieved by the Thurner map at national level (29%) and the Barredo map at sub-national level (36%), but the differences among the maps were not particularly marked nor consistent among the statistics. When compared to the total stocks, the accuracy statistics for mean biomass density were comparable in terms of relative error but lower in terms of variance explained (r^2^), likely due to the smaller range of values for mean biomass compared to those for total stock.

### 3.3 Maps assessment using the harmonized plots

**Fig. 5:**
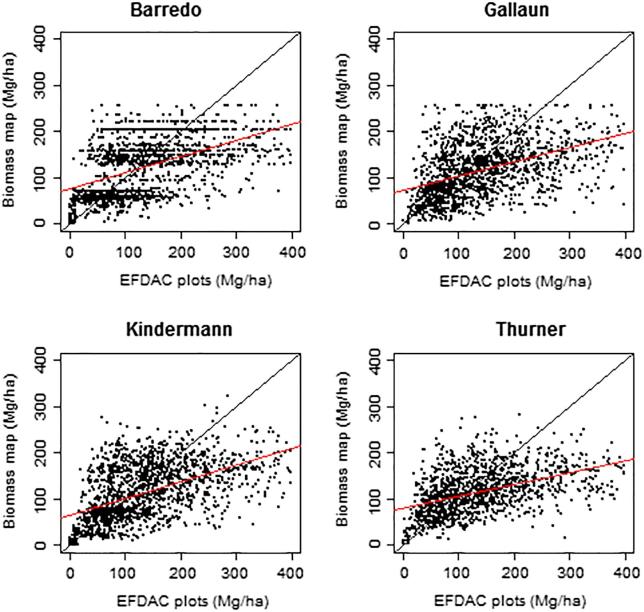
Comparison of the biomass maps with the selected EFDAC plot dataset. The red lines represent the linear regressions between the two datasets and the black lines represent the 1:1 line. (For interpretation of the references to color in this figure legend, the reader is referred to the web version of this article.)

The assessment of the biomass maps using the selected EFDAC field plots confirmed that, as indicated by the statistics, all four maps tended to underestimate the biomass density in comparison with the EFDAC estimates (Table 6). The assessment results were similar among the maps, with the Barredo map presenting slightly lower relative error and the Gallaun map lower absolute error. The maps bias was between −5 and −23 Mg ha^−1^, the RMSE between 78 and 80 Mg ha^−1^ and the relative RMSE between 58% and 67%. Hence, at pixel level the bias was smaller but the absolute and relative errors were substantially higher that those reported at national and sub-national levels (Fig. 5).

**Table 6:** Assessment of the biomass maps using the selected EFDAC plot dataset.

	Barredo	Gallaun	Kindermann	Thurner
N. plots	1556	1078	1561	1272
Bias (Mg ha^−1^)	−14	−5	−22	−23
r^2^	0.25	0.17	0.27	0.21
RMSE (Mg ha^−1^)	78	79	79	80
Rel RMSE (%)	58%	67%	59%	60%

The statistics by biomass range showed that all maps tended to overestimate biomass in the range 0–100 Mg ha^−1^ and underestimate it at higher values. The maps presented lower absolute errors but higher relative errors in the range 0–100 Mg ha^−1^. Interestingly, the maps accuracy was highest in the range 100–200 Mg ha^−1^, where the maps bias was between −9 and −21 Mg ha^−1^, the RMSE between 53 and 58 Mg ha^−1^ and the relative RMSE between 37% and 40% (Fig. 5, Table 7).

**Table 7:** Assessment of the biomass maps by biomass range using the selected EFDAC plot dataset.

	Biomass bin	Bias (Mg ha^−1^)	RMSE (Mg ha^−1^)	Rel. RMSE (%)	N. plots
Barredo	0–100	30	59	105	638
Gallaun		40	64	122	513
Kindermann		23	54	95	644
Thurner		30	51	90	513

Barredo	100–200	−9	57	39	581
Gallaun		−9	54	37	375
Kindermann		−17	58	40	581
Thurner		−21	53	37	481

Barredo	200–300	−85	100	41	249
Gallaun		−100	113	46	142
Kindermann		−92	108	44	249
Thurner		−103	113	47	203

Barredo	300–400	−173	180	52	88
Gallaun		−184	192	56	48
Kindermann		−178	185	54	87
Thurner		−192	198	57	75

These results were obtained using the selected plots, which were about 5–7% of the complete field dataset and ranged from 1078 plots for the Gallaun map to 1561 plots for the Kindermann map. Nonetheless the small size, the selected plots represented well the complete dataset both in terms of spatial coverage and frequency distribution of biomass (Figs. A.3 and A.4). Furthermore, the maps accuracy obtained using the selected plots was considerably higher than that obtained with all available plots. On average, by using the selected plots the maps bias decreased by 54%, the r^2^ increased by 51%, the RMSE decreased by 24% and the relative RMSE decreased by 34% compared to the results obtained using all plots without any screening (Table A.1). Hence, even though the plot selection reduced considerably the amount of data available for the assessment, it also provided more reliable accuracy results because it removed the errors only due to the spatial mismatch between the plots and the pixels.

## 4 Discussion

### 4.1 Towards fully harmonized biomass statistics for Europe

European NFIs have long tradition and are recognized as the most reliable source of information about the state of the European forest. However, each NFI uses country-specific design and definitions, which introduce substantial uncertainty when the national data are compiled for regional assessments (Neumann et al., 2016). The regional and global statistics such as in FAO FRA and in FOREST EUROPE are based on common forest definitions but do not consider the differences in biomass definition and estimation. We found that the difference of total biomass stock between the national and harmonized statistics was relatively small at European scale (3.8%), but it was substantial at national scale for 14 countries. Hence, the harmonized EFDAC biomass statistics used in this study represent a major step ahead towards a consistent and accurate assessment of forest biomass resources in Europe.

We note that the EFDAC statistics use NFI data referring to different years (2002–2013) and their integration may be further improved by temporal harmonization. Even though the data acquisition from the NFIs necessarily follow the country circumstances, a posteriori data synchronization may be obtained using modelling approaches such as the Carbon Budget Model (Jonsson et al., 2017, Pilli et al., 2016), and thus obtain national statistics referring to the same year across Europe. Instead, the temporal mismatch between the NFI plots and the biomass maps was taken into account and removed in this study using the IPCC biomass increments by ecozone. This step may be further refined by using country-, age- and/or species-specific biomass increment values, which currently are not available for most countries but could be derived from forest growth models, such as the Global Forest Model (Kindermann et al., 2013).

We stress that, when assessing maps with ground observations, it is essential to carefully remove the plots not representative of the map cells with which they are compared, and for this purpose we employed an innovative approach based on tree cover variability and histogram normalization. Even though tree cover is only a proxy of biomass, using stringent screening criteria allowed to identify homogeneous forest areas where it is likely that the biomass variability is minimized, hence removing most differences due to mismatches in spatial resolution. The effectiveness of the selection procedure was reflected by the improved maps performance compared to the results without selection. However, our results may still be affected by the local scale biomass variability not captured in our procedure, and more robust results would be obtained with fully comparable data. Such data can be obtained by acquiring ground measurements using a sampling design more suitable to upscaling and extrapolating to larger areas or by using airborne lidar data to identify areas with homogeneous forest structure, as well as by producing biomass maps with higher spatial resolution.

### 4.2 Performance of the biomass maps

The performance of the biomass maps was assessed at regional, national, sub-national and pixel levels using the harmonized statistics and plots. Interestingly, even though the biomass maps were produced with different data and approaches, the results indicate that all maps tended to underestimate biomass density compared to the NFI data. More precisely, the maps overestimated at low biomass (0–100 Mg ha^−1^) and underestimated at medium – high biomass density (>100 Mg ha^−1^). This trend can be due to the following reasons.

The Barredo and Kindermann map downscaled national reference data (as reported to the FAO) using spatial maps and therefore the total country stocks were unbiased but the mean densities assessed in dense forest areas were lower than the reference values. The Barredo map was mainly driven by the use of mean reference values by forest type, which likely caused the overestimation at low biomass and the underestimation at high biomass as mean values cannot fully represent the data variability. Instead, the Kindermann map downscaled the national stocks using assumptions aimed to depict the biomass distribution at global scale, which may not fully fit the biomass distribution in Europe. For instance, this map assumed that managed forests store half of the biomass of undisturbed forests, which may underestimate the stocks of central European forests where most managed stands can reach high biomass densities before logging (Neumann et al., 2016).

The Gallaun and Thurner maps were mainly driven by models based on remote sensing data, and their uncertainty is likely due to the limited sensitivity of satellite sensors to variations in canopy height and tree diameter. More specifically, underestimation may be due to the saturation of optical and short-wavelength radar sensors (i.e., MODIS and ASAR) at high biomass in dense forest, while overestimation may occur in open forests or young stands having relatively wide or closed canopy but small tree diameter and height (Avitabile et al., 2012, Santoro et al., 2015). Furthermore, the Gallaun map limited the biomass estimates to the maximum value of 256 Mg ha^−1^ at 500 m resolution while central European mature forests may reach higher densities.

The maps uncertainty is also due to the coarse spatial resolution of the satellite data, which often results in mixed pixels where a similar signal may correspond to vegetation types having different biomass density (Avitabile et al., 2016). In addition, it was shown that the map errors tend to increase when the calibration data have a size that is much smaller than the map cells (Réjou-Méchain et al., 2014).

The accuracy reported by the maps producers, when provided, used statistics and spatial scales not fully compatible with our results but often the estimated errors were similar or lower than those found in this study. Specifically, Thurner et al. (2014) reported errors at national levels and found a relative RMSE of 30–40% for the northern hemisphere and a r^2^ of 0.7 (RMSE of 17.4 Mg ha^−1^) for Europe. Barredo et al. (2012) reported errors at sub-national level and indicated a r^2^ of 0.89 for 30 sub-national units. Gallaun et al. (2010) reported uncertainty at regional level only for the volume map with a mean absolute error of 25 m^3^ ha^−1^ and a correlation coefficient (r) of 0.97. Kindermann et al. (2008) did not report uncertainty statistics. Interestingly, both the Gallaun and Thurner maps also found a small negative bias in their maps due to underestimation at high biomass.

Lastly, it is noted that the estimates of biomass stocks are affected by the choice of the forest mask (Seebach et al., 2012). Existing land cover or forest maps differ considerably for forest definition, thematic content and spatial resolution, and maps with moderate resolution (e.g., 1 km) and mixed classes present large mosaic areas with a fractional coverage of forest, which introduce large variability and uncertainty regarding their biomass density.

## 5 Conclusions

In most European countries there is a long tradition of forest inventory providing reliable and consistent statistics of forest resources that are used for national monitoring purposes. In the last years the need has emerged for a coordinated assessment of forest biomass at European scale to support EU policies and for international reporting purposes. At the same time, a few biomass maps based on remote sensing data have been produced at regional or global scales, but they present divergent estimates and do not provide complete uncertainty information, making it difficult to assess the accuracy of their estimates.

This study presents forest biomass statistics harmonized for biomass definition and estimation for 26 European countries, and uses them to assess existing biomass maps for Europe. Our results show that the published biomass maps tend to underestimate biomass in dense forests and overestimate it in open forests. The mismatch with the reference data is higher at pixel level, indicating that such maps may not be appropriate for local scale applications and suggesting the need for an updated and improved European biomass map.

Despite the limited accuracy reached by existing products, biomass maps remain essential as they provide comprehensive estimates over large areas at a spatial resolution (≤1 km) that opens up the possibility for several applications and value-added products not obtainable by summary statistics. For example, a wall-to-wall forest biomass map may serve as a basis for spatially explicit forest carbon accounting and to initialize forest and landscape dynamics simulators, while its integration with geospatial data such as forest accessibility or terrain conditions would allow to better assess the biomass stocks available for wood supply and related extraction costs (Alberdi et al., 2016).

Even though Europe will not be covered by the upcoming ESA BIOMASS satellite (Carreiras et al., 2017), new biomass maps with improved accuracy and higher spatial and temporal resolutions will be produced in the coming years thanks to better data acquired by dedicated satellite missions (e.g., the GEDI and NISAR missions) and larger availability of airborne lidar data (Goetz et al., 2015, Morton, 2016), improved modelling approaches (Santoro et al., 2017) and new data fusion techniques (Avitabile et al., 2016, Goetz et al., 2015). Furthermore, time-series data recently acquired by the ESA Sentinel 1 and 2 missions and related products such as the Copernicus high resolution forest layers providing recent and detailed information on forest extents and properties (Probek et al., 2014) will contribute to obtain improved and up-to-date biomass estimates. In this context, the role of the European NFIs will not decrease but instead it will become even more important because they will provide the key reference data to calibrate and assess the accuracy and reliability of the new biomass products.
