# Supplementary material for: An assessment of forest biomass maps in Europe using harmonized national statistics and inventory plots
Source: For Ecol Manage. 2018 Feb 1;409:489–98. doi: 10.1016/j.foreco.2017.11.047 (PMC5806600; doi:10.1016/j.foreco.2017.11.047)
Supplement: Supplementary data 1 [file mmc1.docx]

# Appendix A. Supplementary Information

Figure A.1: Total biomass stock per country, using the national or harmonized definitions in combination with the national or common estimators. The error bars represent the sampling errors and do not include the uncertainties in the biomass estimation from tree parameters (data source: Henning et al., 2016; Korhonen et al., 2014)

Figure A.2: Mean biomass density per country, using the national or harmonized definitions in combination with the national or common estimators. The error bars represent the sampling errors and do not include the uncertainties in the biomass estimation from tree parameters (data source: Henning et al., 2016; Korhonen et al., 2014)


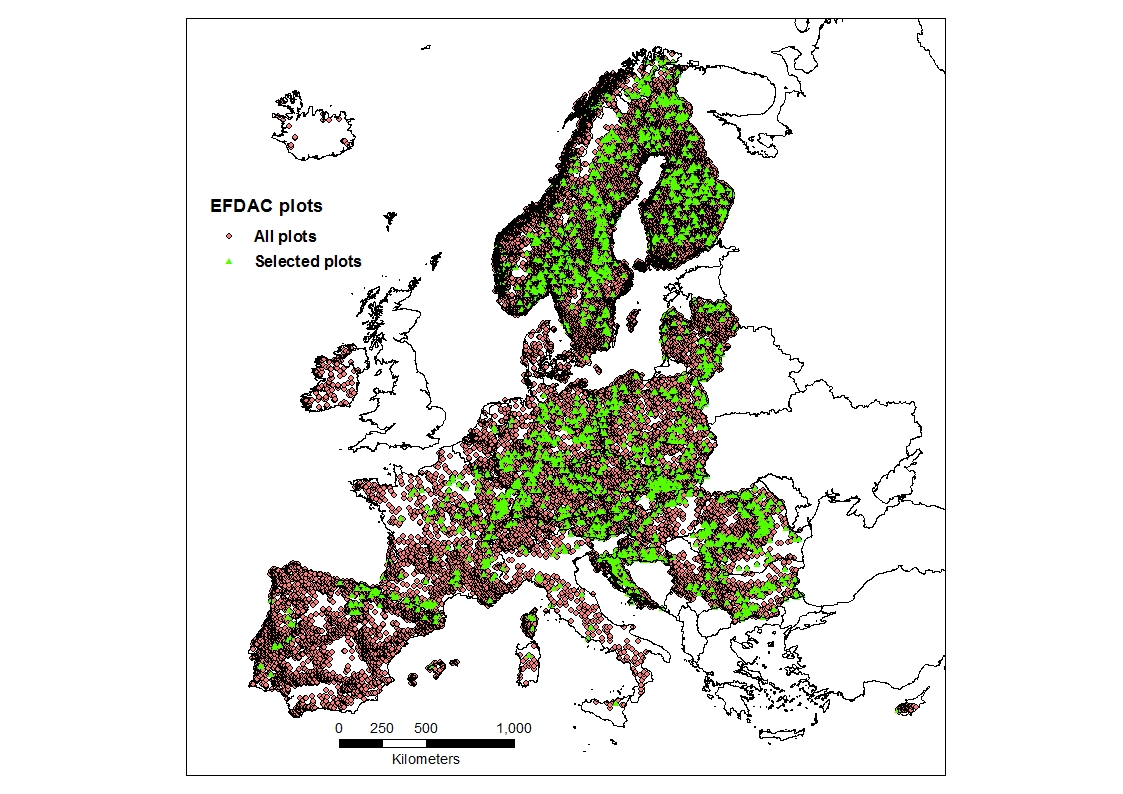
Figure A.3: Spatial distribution of all and selected EFDAC plots


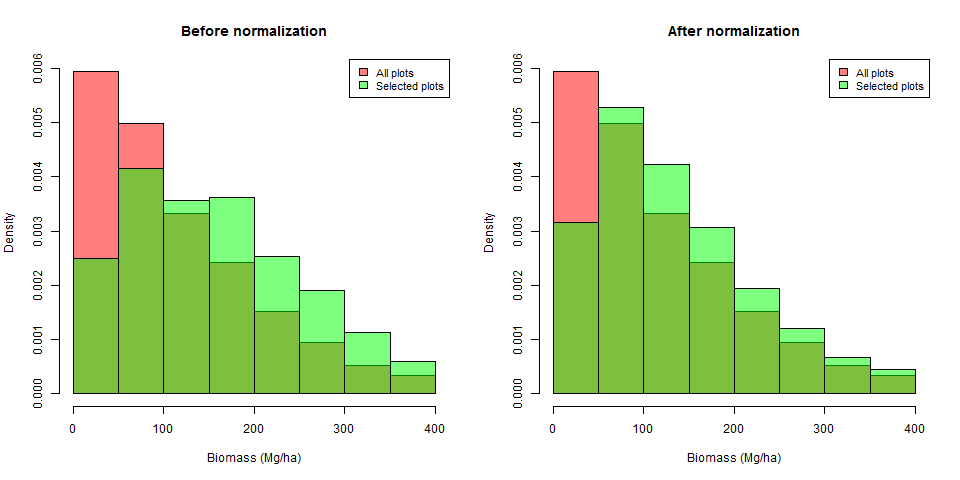
Figure A.4: Relative frequency distribution (density) of all and selected EFDAC plots by biomass bin before (left) and after (right) the histogram normalization

Table A.1: Assessment of the biomass maps using all harmonized EFDAC plots without selection procedures

|  | Barredo | Gallaun | Kindermann | Thurner |
| --- | --- | --- | --- | --- |
| N. plots | 20,321 | 18,126 | 21,388 | 13,612 |
| Bias (Mg ha^-1^) | -42 | -29 | -50 | -27 |
| r^2^ | 0.15 | 0.12 | 0.14 | 0.19 |
| RMSE (Mg ha^-1^) | 107 | 100 | 110 | 99 |
| Rel RMSE (%) | 90 | 103 | 93 | 83 |
